# Chemical Composition of Fresh Leaves Headspace Aroma and Essential Oils of Four Coriander Cultivars

**DOI:** 10.3389/fpls.2022.820644

**Published:** 2022-02-17

**Authors:** Sunjeet Kumar, Raza Ahmad, Sidra Saeed, Muhammad Azeem, Raimondas Mozūraitis, Anna-Karin Borg-Karlson, Guopeng Zhu

**Affiliations:** ^1^Key Laboratory for Quality Regulation of Tropical Horticultural Crops of Hainan Province, School of Horticulture, Hainan University, Haikou, China; ^2^Department of Biotechnology, COMSATS University Islamabad, Abbottabad, Pakistan; ^3^Department of Chemistry, COMSATS University Islamabad, Abbottabad, Pakistan; ^4^Department of Zoology, Stockholm University, Stockholm, Sweden; ^5^Laboratory of Chemical and Behavioral Ecology, Nature Research Centre, Institute of Ecology, Vilnius, Lithuania; ^6^Department of Chemistry, School of Engineering Sciences in Chemistry, Biotechnology and Health, Royal Institute of Technology, Stockholm, Sweden; ^7^Department of Chemical Engineering, Mid Sweden University, Sundsvall, Sweden

**Keywords:** aroma, coriander leaves, essential oil, free radical scavenging, gas chromatography-mass spectrometry, headspace, solid phase micro extraction

## Abstract

Aroma is one of the key food characteristics determining consumers’ perception and acceptability of products. *Coriandrum sativum* L. is an aromatic herb commonly used as a food additive and taste enhancer. Besides the culinary applications, coriander is also used in traditional medicine, cosmetics, and the food industry. In this study, we aimed to determine aroma composition of fresh chopped leaves and essential oils extracted from the leaves of four coriander cultivars. The essential oils were extracted from the fresh leaves using steam distillation and volatile aroma components were collected from the headspace by solid phase micro extraction technique. Analyses were carried out by gas chromatography-mass spectrometry. Free radical scavenging activity of essential oils was determined by using 2,2-diphenyl-1-picrylhydrazyl assay. The essential oils were also investigated for their anti-microbial potential. The aroma of freshly chopped coriander leaves was characterized by thirteen compounds, including six aldehydes, four alcohols, one ester and one hydrocarbon. The essential oils were comprised of twenty-seven compounds, where (*E*)-2-decenal, decanal, (*E*)-2-dodecenal and (*E*)-2-tetradecenal were the main components in all cultivars. Free radical scavenging activity of the essential oil samples was in the range of 6–15%. The essential oils of Desi and Hybrid cultivars exhibited least minimum inhibitory concentration (MIC) against all tested bacterial strains. Fresh green leaves of the Desi and Peshawari cultivars were found to be the richest in six carbon chain length alcohols and acetates, which are important constituents of the aroma giving a characteristic odor referred to as the “green note.” The Hybrid cultivar showed the highest free radical scavenging activity, bearing the highest amount of antioxidants. The study revealed that the fresh leaves HS aroma of Desi and Hybrid cultivars were different, however, their essential oils possessed almost similar chemistry and anti-bacterial activity.

## HIGHLIGHTS

- Aroma of chopped coriander leaves was characterized by six aldehydes, four alcohols, one hydrocarbon and an ester.
- Six carbon chain alcohols and acetates gave a characteristic odor referred to as the “green note.”
- Essential oils from the fresh coriander leaves were comprised of 27 compounds.
- Free radical scavenging activity of the essential oil samples was in the range of 6–15%.
- Desi and Hybrid cultivars exhibited least MIC against bacteria compared to Peshawari and Irani cultivars.

## Introduction

Aroma is one of the most important food sensory characteristics determining whether or not a product will be accepted by a potential consumer (Mahony, 1990). Humans can sense more than 7,000 volatile compounds through the use of 347 olfactory receptors (Goff and Klee, 2006). Aroma is a complex mixture of a large number of volatile compounds and its specificity depends upon the combination of various volatile compounds, their relative concentration as well as the perception threshold of an individual volatile compound (Tucker, 1993). Identifying key volatile aroma metabolites that represent the unique character of the food products is essential, as it provides the principal sensory identity and characteristic flavor of products, which could be used to monitor and improve sensory quality of a food after processing steps (Cheong et al., 2010). Herbs and spices are rich sources of phytochemicals and are commonly used to improve aroma and prolong the shelf-life of food products (Embuscado, 2015).

*Coriandrum sativum* L. (Apiales: Apiaceae) is one of the oldest aromatic herbs that has been used as a food additive and for medicinal purposes for over 3,000 years (Sahib et al., 2013). It originated from the Mediterranean region but has been well domesticated in large parts of Asia, Europe and North America (Paarakh, 2009). Coriander is commonly used to enhance the flavor of different cuisines, sauces and salads. All of the aerial parts of the coriander plant are consumed; the leaves and the stem are used in salads or for garnishing dishes, whereas the seeds are consumed as a spice, enhancing the flavor of cooked and raw food (Singletary, 2016). Some industries related to confectionary products also use coriander as a taste enhancer.

Besides for aromatic purposes, coriander is also a good source of vitamins, minerals, dietary fiber and other trace elements (Prachayasittikul et al., 2018), which can help to meet the nutritional requirement of the people living in developing countries (Bhat et al., 2014). Coriander is used as herbal medicine to treat a number of ailments such as stomach problems, dysentery, vomiting, jaundice, cough and diarrhea. It also possesses antirheumatic, antidiabetic, cytotoxicity, antibacterial, antifungal, and antioxidant activities (Khan and Khatoon, 2008; Paarakh, 2009). The aldehydes present in coriander are important for curing toothache, measles, neurasthenic and for protection against cancer (Deng et al., 2003). Coriander essential oil is highly important due to its use in the cosmetic industry for making perfumes, different types of skin protecting products and creams (Laribi et al., 2015).

Most of the previous studies presented the chemical composition of coriander essential oils obtained from seeds (Bandoni et al., 1998; Eikani et al., 2007; Msaada et al., 2007; Bhuiyan et al., 2009; Zoubiri and Baaliouamer, 2010), consisting of large amounts of both enantiomers of linalool, whereas some publications reported chemical analysis of essential oils extracted from fresh coriander leaves (Chung et al., 2012; Satyal and Setzer, 2020). There are two studies from Pakistan where the chemical composition of seed (Anwar et al., 2011) and leaves’ essential oils (Shahwar et al., 2012) was investigated. However, there are only a few literature sources available on the headspace aroma composition of fresh coriander leaves. Studies from China, Japan, United States and Italy report the chemical composition of green coriander leaves aroma (Fan and Sokorai, 2002; Deng et al., 2003; Kohara et al., 2006; Carrubba et al., 2007).

In South Asian cultures, coriander leaves are used in raw form or cooked in hot dishes. Hence, considering the effect of heat on the aroma composition of coriander leaves, we compared the aroma composition of both fresh coriander and essential oils from four coriander cultivars leaves. Up to date, there is no study where the same cultivars of coriander were examined for both the headspace aroma and the essential oil composition.

Coriander is also valued for its antioxidants, which prevent lipids, essential fatty acids and vitamins from oxidative degradation. To evaluate the anti-oxidant potential of the essential oil samples, we measured free radical scavenging activity by using 2,2-diphenyl-1-picrylhydrazyl (DPPH) assay. Coriander is widely used in traditional medicines, pharmaceuticals, and food industries (Burdock and Carabin, 2009). The fresh leaves essential oils of coriander cultivars were also tested for their anti-microbial potential against pathogenic bacterial strains.

## Materials and Methods

### Coriander Seed Collection and Cultivation

Four coriander cultivars commonly grown in the South Asia and Middle East (Desi, Peshawari, Irani, and Hybrid) were used in this study. The certified seeds of these cultivars were obtained from different sources, such as (1) the seeds of Desi cultivar (also known as Dilpazeer) and Hybrid cultivar were kindly provided by Ayub Agricultural Research Institute, Faisalabad, Pakistan, where the Hybrid cultivar seeds were actually imported from Italy (Suba Seeds Company SPA, Italy). (2) The certified seeds of Irani and Peshawari cultivars were purchased from North South Seeds Pvt. Ltd., Peshawar, Pakistan. The seeds of the four coriander cultivars were sown in small plots (200 cm × 150 cm) during the first week of March at the experimental field. The experimental plots were treated in the same way to ensure optimum growth of plants thus regularly irrigated, however, no fertilizer was used. After 2 months of growth, the aerial parts of the plants were plucked and used for aroma headspace (HS) chemical analysis and essential oil extraction. It is pertinent to mention that all the plants were at same physiological stage and there were no inflorescence.

### Collection of Headspace Volatiles From Coriander Leaves

Fresh green coriander leaves were harvested from the plants and about 2 g of knife chopped leaves were placed in a 100 mL Erlenmeyer flask (E-flask). The opening of the E-flask was closed with aluminum foil that was further tightened with a rubber band. Prior to the collection of volatiles from the coriander leaves, the HS in the flask was equilibrated for 60 min at room temperature (25 ± 2°C). Solid phase micro extraction (SPME) fiber was used for the collection of volatiles. The SPME fiber consisted of polydimethylsiloxane/divinylbenzene (PDMS/DVB) coating on a StableFlex^TM^ fiber (Supelco, PA, United States). The SPME fiber was fitted into a SPME holder (Supelco, PA, United States) to be used as a normal GC syringe for manual injection. The SPME fiber was conditioned as advised by the manufacturer under a stream of helium at 250°C for 30 min prior to its first use for collection of volatiles. After conditioning/cleaning, the needle of the SPME was introduced into the HS of E-flask through a pin hole in the aluminum foil covering the flask (Azeem et al., 2015). The SPME fiber was exposed to the HS of E-flask containing chopped coriander leaves for 45 min. The SPME fiber was pulled back immediately after the collection of volatiles and the SPME needle was injected into a gas chromatograph (GC) injector. The SPME fiber was exposed in the GC injector for 5 min for thermal desorption and cleaning. At least three replicates of each coriander variety were employed under the same conditions.

### Extraction of Essential Oil

The essential oil from the fresh coriander leaves was obtained through steam distillation. Freshly harvested coriander leaves (500 g) were subjected to steam distillation in a stainless steel distillatory and the distillate was collected for 4 h (Azeem et al., 2019, 2022). The collected distillate was extracted three times with 70 mL of HPLC grade *n*-hexane (Daejung, South Korea). The extracted essential oil solution was dried by adding a small amount of anhydrous MgSO_4_ (Daejung, South Korea) to remove any traces of water, and filtered through Whatman filter paper. Subsequently, the extracts were concentrated by using a rotary evaporator at 25°C under reduced pressure. The pure essential oils were weighed to calculate their percentage yields (w/w). Essential oil from each coriander cultivar was extracted in duplicated manner. The essential oil samples were stored at −20°C, until used in further experiments.

### Separation and Identification of Volatile Compounds

PerkinElmer Clarus 500 gas chromatograph (Perkin Elmer, United States) equipped with flame ionized detector (FID) was used for the separation and quantification of volatile compounds collected from the HS of the coriander leaves by SPME. The GC was equipped with a split/splitless injector operating in splitless mode for 60 s. Nitrogen gas, with a constant flow of 1 mL/min through the column, was used as a carrier gas. Elite-5 (Perkin Elmer, United States) capillary column with a 30 m length, 0.53 mm internal diameter and 1.5 μm film thickness was installed in the GC. The temperature program of the GC oven was set at 40°C for 30 s, then ramped at the rate of 5°C per min up to 180°C where it was kept isothermal for 10 s. Afterward, it was additionally ramped up at the rate of 15°C per min up to 230°C. Finally, the oven temperature was kept isothermal at 230°C for 13 min. The injector and detector temperature were set isothermal at 225 and 250°C, respectively. The retention index of the separated compounds was calculated using the retention time data of C_8_–C_24_ *n*-alkanes mixture (Sigma-Aldrich Sweden) obtained under the same chromatographic parameters as those used for the analysis of SPME collected coriander volatile compounds. Chromatographic records were analyzed by using Total Chrome Work Station PerkinElmer, version 6.3.1 software.

Hewlett Packard 6890N GC coupled with Hewlett Packard 5973 mass spectrometer (MS) (Agilent Technologies Inc., United States) was used to separate and identify compounds sampled from coriander HS and essential oils. The GC was equipped with a HP-5 capillary column with 30 m length, 0.25 mm internal diameter and 0.25 μm film thickness (Agilent Technologies, United States). The carrier gas was high purity helium with a flow rate of 1 mL/min. The GC oven program was the same as that used for GC-FID. The GC was equipped with a split/splitless injector operating in splitless mode for 60 s. The injector and the transfer line temperatures were set constant at 225 and 235°C, respectively. The mass spectra of separated compounds were obtained at 70 eV with a scan range of 30–400 amu. The ion source of the mass spectrometer was set at 180°C. All compounds were identified by comparing their mass spectra and retention indexes with those present in the NIST-2008 MS library, afterward comparing with published retention index values and finally with those of authentic standards using MSD Productivity ChemStation (v.02.01.1177). The composition of essential oils was reported as a relative percentage of the total peak area of all identified peaks. Essential oils extracted from each coriander cultivar were analyzed three times and mean percentage composition was reported.

### 1,1-Diphenyl-2-Picrylhydrazyl Scavenging Activity of Fresh Coriander Leaves’ Essential Oils

1,1-Diphenyl-2-picrylhydrazyl (DPPH) is a stable free radical compound with an unpaired valence electron present at one atom of nitrogen bridge. This compound is used to access the free radical scavenging activity of any compound or biological extract (Eklund et al., 2005). The DPPH scavenging activity of fresh leaves’ essential oils was determined using the method described by Aoshima et al. (2004) with some modification. Briefly, 2.5 mL solution of DPPH (0.1 mM in methanol) was mixed with 1 mL methanolic solution of essential oil (100 μg/mL) in a falcon tube. The falcon tube was covered with aluminum foil to avoid light exposure. The contents of falcon tubes were vortexed for 30 s and incubated in the dark for 30 min at 25°C. Three replicates were used for each essential oil. A gallic acid solution (100 μg/mL) was used as a positive control. The discoloration of the DPPH (dark purple) was determined by measuring absorbance at 517 nm on a double beam spectrophotometer. The percentage of DPPH inhibition was calculated by using the following correlation:% DPPH Inhibition = (Absorbance of blank - Absorbance of sample) × 100/Absorbance of blank.

### Anti-bacterial Activity

Four bacterial strains namely *Escherichia coli* ATCC 25922, *Pseudomonas aeruginosa* ATCC 9027, *Bacillus subtilis* ATCC 6633, and *Staphylococcus aureus* ATCC 6538 were used to investigate the anti-bacterial potential of fresh coriander leaves essential oils. Freshly cultured bacterial cells were suspended in sterilized saline and the optical density of bacterial suspension was adjusted to 0.5 at 600 nm. The bacterial suspension was further diluted to get 10^4^ CFU/mL that was used as seed culture for antibacterial bioassay. The minimum inhibitory concentration (MIC) of coriander fresh leaves essential oils was determined by using the broth microdilution method (Vu et al., 2016). Briefly, twofold serial dilutions of each coriander variety essential oils prepared in DMSO was added to the wells of a 96-well plate containing 100 μL nutrient broth (NB) medium inoculated with bacterial suspension (10^4^ CFU/mL). The final concentrations of the pure essential oils were ranged from 250 to 4,000 μg/mL. DMSO was used as negative control and to make different dilutions of essential oils but its concentration in each bacterial suspension was never more than 1% that did not affect bacterial growth. After incubation at 37°C for 24 h, the MIC was determined as the lowest concentration that completely inhibited the growth of the bacteria. At least three replicates of each dilution of essential oils were employed against each tested bacteria.

### Statistical Analysis

One-way ANOVA with *post hoc* Tukey test was used to find the significant differences between the free radical scavenging activity of different coriander cultivar essential oils and gallic acid. The same test was employed to find significant difference between the relative abundance of different compounds found in the HS and essential oils of four coriander cultivars independently. The statistical analysis was carried out using SPSS (IBM, United States) computer software.

## Results

### Chemical Composition of Headspace Aroma of Fresh Coriander Leaves

The SPME collected HS aroma of freshly plucked and chopped coriander leaves revealed the presence of thirteen compounds (Table 1). (*Z*)-3-Hexenyl acetate was the major compound found in all four coriander cultivars ranging from 66.12 ± 1.1% to 77.12 ± 1.2% of the total aroma detected by HS-SPME and GC-FID. The proportion of this compound was higher (*P* < 0.05) in the Irani and Peshawari cultivars compared to Desi and Hybrid cultivars (Table 1). The Hybrid cultivar exhibited the lowest proportion of (*Z*)-3-hexenyl acetate compared to other three cultivars. (*Z*)-3-Hexenol was observed as the second most abundant compound in the HS of Desi, Irani and Peshawari cultivars, however, the relative abundance of this compound was significantly different (*P* < 0.05) in four cultivars. The proportion of (*Z*)-3-hexenol in the Desi and Peshawari cultivars HS aroma was 13.13 ± 0.6% and 10.12 ± 0.5% whereas in the Hybrid and Irani cultivars it was only 6.04 ± 0.3% and 7.25 ± 0.3% (Table 1). Most of the identified compounds were present in the HS aroma of all coriander cultivars except (*E*)-2-hexenol, which was present only in the Hybrid cultivar, as well as 1-nonanol, which was present in trace amount only in the Hybrid and Irani cultivars’ HS (Table 1).

**TABLE 1:** Percentage composition of compounds detected in the headspace of fresh coriander leaves of four coriander cultivars.

**Compound name**	**RI**	**Desi**	**Hybrid**	**Irani**	**Peshawari**
**(*Z*)-3-Hexenol**	**858**	**13.13** ± **0.6**^a^	**6.04** ± **0.3**^d^	**7.25** ± **0.3**^c^	**10.12** ± **0.5**^b^
1-Hexanol	870	0.99 ± 0.1^a^	0.25 ± 0.1^b^	0.33 ± 0.1^b^	0.26 ± 0.1^b^
(*E*)-2-Hexenol	883	−	1.07 ± 0.1	−	−
**Nonane**	**900**	**2.63** ± **0.2**^c^	**8.06** ± **0.5**^a^	**3.62** ± **0.3**^b^	**0.95** ± **0.2**^d^
**(*Z*)-3-Hexenyl acetate**	**1,009**	**71.7** ± **1.5**^b^	**66.12** ± **1.1**^c^	**77.12** ± **1.3**^a^	**75.63** ± **1.4**^a^
1-Nonanol	1,165	−	0.15 ± 0.1^a^	0.08 ± 0.1^a^	−
**Decanal**	**1,208**	**0.62** ± **0.1**^b^	**2.17** ± **0.2**^a^	**0.58** ± **0.1**^b^	**0.41** ± **0.1**^c^
**1-Decanol**	**1,274**	**2.16** ± **0.3**^b^	**3.74** ± **0.5**^a^	**2.77** ± **0.5**^b^	**4.14** ± **0.4**^a^
Dodecanal	1,412	0.43 ± 0.2^b^	1.59 ± 0.2^a^	0.58 ± 0.1^b^	0.57 ± 0.1^b^
**(*E*)-2-Dodecenal**	**1,470**	**1.29** ± **0.4**^c^	**1.98** ± **0.5**^ab^	**2.14** ± **0.2**^a^	**1.64** ± **0.3**^bc^
(*E*)-2-Tridecenal	1,572	0.2 ± 0.1^b^	0.45 ± 0.1^a^	0.19 ± 0.1^b^	0.21 ± 0.1^b^
**(*E*)-2-Tetradecenal**	**1,676**	**1.74** ± **0.4**^a^	**2.28** ± **0.2**^a^	**1.88** ± **0.3**^a^	**1.33** ± **0.1**^b^
(*Z*)-9-Hexadecenal	1,777	0.41 ± 0.1^a^	0.43 ± 0.1^a^	0.42 ± 0.2^a^	0.33 ± 0.1^a^
**Total identified**		**95.3** ± **3.5**	**94.33** ± **3.9**	**96.96** ± **3.5**	**95.59** ± **3.1**

*Retention indexes (RI) of compounds were calculated on a HP-5 gas chromatographic column. Data presented is percentage composition ± standard deviation (n = 3). Different letters in a row indicate significant difference (P < 0.05, ANOVA post hoc Tukey test) between the relative abundance of a compound in four coriander cultivars HS aroma analyzed through HS-SPME GC-FID. Major compounds are presented in bold font.*

### The Yield of Essential Oils

The percentage yield of essential oils extracted from fresh coriander leaves was in the range of 0.027–0.042%. The highest percentage yield was obtained from the Peshawari cultivar, whereas the Desi cultivar showed the smallest yield among all cultivars. The density of the extracted oils ranged from 0.73 to 0.78 g/mL (Table 2).

**TABLE 2:** Percentage yield and density of essential oils extracted from the fresh leaves of four coriander cultivars.

**Coriander cultivars**	**Density g/mL**	Percentage yield*
Desi	0.78	0.027 ± 0.002
Hybrid	0.75	0.030 ± 0.003
Irani	0.79	0.039 ± 0.003
Peshawari	0.73	0.042 ± 0.005

*Percent yield expressed as w/w.

### Chemical Composition of Coriander Leaves’ Essential Oils

Twenty-seven compounds were identified in essential oils extracted from green coriander leaves (Table 3). Most of the identified compounds were present in the essential oils of all four coriander cultivars, except traces of 3-butenyl isothiocyanate which was only present in the Desi cultivar (Table 3). The (*E*)-2-decenal was the most abundant compound in the essential oils of Hybrid, Irani and Peshawari cultivars, constituting 20.1 ± 0.4, 23.4 ± 0.6, and 25.1 ± 0.5% of the essential oil, respectively. This compound was the second most abundant compound (15.7 ± 0.3%) in the Desi cultivar. The relative abundance of (*E*)-2-decenal in all the four cultivars was significantly different (*P* < 0.05) from each other. The most abundant compound of the Desi cultivar was decanal, composing 18.4 ± 0.5% of the essential oil whose relative abundance is similar (*P* > 0.05) to Hybrid cultivar whereas significantly higher (*P* < 0.05) than those of Irani and Peshawari cultivars which are in turn different from each other. Contrary to the Desi cultivar, decanal was the second most abundant compound in the essential oils of the Hybrid, Irani and Peshawari cultivars (Table 3). (*Z*)-3-Hexenyl acetate and 1-octanol were not present in the essential oil of the Hybrid cultivar, whereas their minute amounts were detected in the essential oils of the other three cultivars (Table 3).

**TABLE 3:** Percentage chemical composition of essential oils extracted from fresh coriander leaves of four cultivars through steam distillation.

**Compounds**	**RI**	**Desi**	**Hybrid**	**Irani**	**Peshawari**
3-Butenyl isothiocyanate	978	0.1 ± 0.0	−	−	−
Octanal	999	1.0 ± 0.2^a^	0.6 ± 0.1^b^	0.6 ± 0.1^b^	1.2 ± 0.2^a^
(*Z*)-3-Hexenyl acetate	1,003	0.1 ± 0.0^b^	−	0.1 ± 0.0^b^	0.2 ± 0.0^a^
Eucalyptol	1,028	1.0 ± 0.1^a^	0.3 ± 0.0^b^	0.4 ± 0.0^b^	1.0 ± 0.2^a^
1-Octanol	1,068	0.2 ± 0.0^a^	−	0.1 ± 0.0^b^	0.3 ± 0.1^a^
Linalool	1,098	0.2 ± 0.0^b^	0.2 ± 0.0^b^	0.1 ± 0.0^c^	0.5 ± 0.1^a^
Nonanal	1,101	0.5 ± 0.1^b^	0.4 ± 0.2^b^	0.4 ± 0.1^b^	0.6 ± 0.2^a^
1-Nonanol	1,169	0.3 ± 0.1^a^	0.1 ± 0.0^c^	0.2 ± 0.1^b^	0.2 ± 0.0^b^
4-Terpineol	1,176	0.2 ± 0.0^b^	0.2 ± 0.0^b^	0.3 ± 0.0^a^	0.3 ± 0.0^a^
(*Z*)-4-Decenal	1,194	0.4 ± 0.0^b^	0.4 ± 0.0^b^	0.7 ± 0.1^a^	0.9 ± 0.2^a^
**Decanal**	**1,203**	**18.4** ± **0.5**^a^	**18.8** ± **0.6**^a^	**16.4** ± **0.4**^b^	**14.4** ± **0.3**^c^
(*Z*)-2-Decenal	1,245	0.4 ± 0.0^b^	0.5 ± 0.1^b^	0.6 ± 0.1^a^	0.7 ± 0.1^a^
**(*E*)-2-Decenal**	**1,259**	**15.7** ± **0.3**^d^	**20.1** ± **0.4**^c^	**23.4** ± **0.6**^b^	**25.1** ± **0.5**^a^
**(*E*)-2-Decenol**	**1,267**	**7.2** ± **0.5**^b^	**6.5** ± **0.5**^b^	**7.3** ± **0.3**^b^	**9.3** ± **0.2**^a^
**1-Decanol**	**1,269**	**7.4** ± **0.1**^a^	**6.0** ± **0.4**^b^	**5.3** ± **0.4**^bc^	**4.1** ± **0.5**^c^
Undecanal	1,304	2.0 ± 0.2^b^	1.3 ± 0.1^c^	1.8 ± 0.1^b^	2.6 ± 0.1^a^
**2-Undecenal**	**1,360**	**2.4** ± **0.3**^c^	**1.9** ± **0.2**^c^	**3.3** ± **0.2**^b^	**5.1** ± **0.3**^a^
(*Z*)-2-Undecenol	1,369	0.4 ± 0.0^c^	0.3 ± 0.0^c^	0.5 ± 0.0^b^	0.8 ± 0.1^a^
**Dodecanal**	**1,405**	**5.6** ± **0.2**^a^	**5.0** ± **0.3**^a^	**4.5** ± **0.2**^b^	**4.20** ± **0.3**^b^
**(*E*)-2-Dodecenal**	**1,464**	**12.6** ± **0.3**^b^	**13.7** ± **0.4**^a^	**13.2** ± **0.5**^a^	**12.9** ± **0.3**^b^
(*Z*)-2-Dodecenol	1,469	1.6 ± 0.3^a^	1.3 ± 0.4^a^	1.2 ± 0.2^ab^	1.0 ± 0.1^b^
Tridecanal	1,507	0.4 ± 0.0^a^	0.4 ± 0.0^a^	0.3 ± 0.0^b^	0.4 ± 0.0^a^
(*E*)-2-Tridecenal	1,565	0.7 ± 0.1^b^	0.8 ± 0.2^b^	0.8 ± 0.1^b^	1.2 ± 0.1^a^
Spathulenol	1,581	1.2 ± 0.1^a^	1.3 ± 0.2^a^	1.4 ± 0.2^a^	1.3 ± 0.1^a^
Globulol	1,587	1.1 ± 0.2^a^	1.1 ± 0.1^a^	1.2 ± 0.2^a^	1.2 ± 0.2^a^
Tetradecanal	1,609	2.2 ± 0.1^a^	1.9 ± 0.2^a^	1.8 ± 0.1^ab^	1.7 ± 0.1^b^
**(*E*)-2-Tetradecenal**	**1,670**	**12.6** ± **0.3**^a^	**12.9** ± **0.6**^a^	**11.1** ± **0.3**^b^	**9.1** ± **0.2**^c^
**Total identified**		**95.9** ± **4.5**	**96.2** ± **4.2**	**96.8** ± **3.9**	**99.7** ± **3.1**

*The retention indexes (RI) of compounds were calculated on a HP-5 GC column. Data presented is percentage composition ± standard deviation (n = 3). Different letters in a row indicate significant difference (P < 0.05, ANOVA post hoc Tukey test) between the relative abundance of a compound in four coriander cultivars essential oils analyzed on GC-MS. Major compounds are presented in bold font.*

### 1,1-Diphenyl-2-Picrylhydrazyl Scavenging Activity of Essential Oils

**FIGURE 1:**
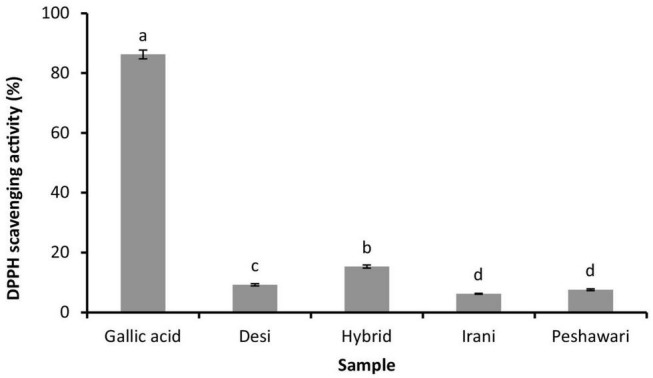
Percent DPPH scavenging activity of essential oils from fresh leaves of four coriander cultivars and gallic acid (positive control). Data are expressed as the mean activity ± standard deviation (*n* = 3). The bars labeled with different letters are significantly different (*P* < 0.05) from each other when data analyzed by ANOVA *post hoc* Tukey test.

The essential oil of the Hybrid cultivar leaves showed the highest DPPH scavenging activity (*P* < 0.05) compared to the other cultivars. The Irani and Peshawari cultivars exhibited similar activity (*P* > 0.05), ranking lowest among all tested essential oils (Figure 1).

### Minimum Inhibitory Concentration

All the essential oils showed anti-bacterial activity against all test bacteria but exhibited different MIC value. The most active essentials were from Desi and Hybrid cultivars that showed 500 μg/mL MIC against all bacteria. Irani and Peshawari cultivars essential oils showed least activity against *B. subtilis* and *E. coli* compared to other two cultivar essential oils (Table 4).

**TABLE 4:** Minimum inhibitory concentration (MIC) of fresh leaves essential oils of four coriander cultivars.

**Cultivars**	**MIC (μ g/mL)**			
	***B. subtilis***	***E. coli***	***P. aeruginosa***	***S. aureus***
Desi	500	500	500	500
Hybrid	500	500	500	500
Irani	1,000	1,000	500	500
Peshawari	1,000	1,000	500	500

## Discussion

The HS compositions of fresh chopped coriander leaves have been previously reported from Italy, China, and United States. We found that the HS of fresh chopped coriander leaves of four cultivars commonly grown in the South Asia and Middle East comprised of six aldehydes, five alcohols, and one of each acetate and hydrocarbon. Among them, (*Z*)-3-hexenyl acetate was the major constituent in all four cultivars, ranging from 66.12 ± 1.1% to 77.12 ± 1.2% of the total aroma volatiles. Other major compounds were (*Z*)-3-hexenol, 1-decanol, (*E*)-2-dodecenal, nonane and (*E*)-2-tetradecenal. The results of the current study are similar to those reported by Carrubba et al. (2007), revealing (*Z*)-3-hexenyl acetate as a major compound (50–70%) along with (*E*) 2-decenal and 1-decanol in the HS of coriander leaves.

A study from China showed a more diverse composition of HS aroma of coriander stems and leaves, reporting (*E*)-2-tridecenal, 1-decanol, (*E*)-2-dodecenal and (*E*)-2-decenol as major compounds (Deng et al., 2003). Thirteen compounds including decanal 51.5%, (*E*)-2-decenal 31.6% and (*E*)-2-dodecenal 5% were identified in the HS of coriander leaves homogenate, made in calcium chloride from United States (Fan and Sokorai, 2002).

In these studies, the sampling of volatiles was conducted under different conditions than in current study, which complicates the comparison of data. In the current and Carrubba et al. (2007) studies, the volatiles from fresh leaves were collected at room temperature using PDMS/DVB and PDMS types of SPME fiber, respectively, and the analysis was performed on HP-5 column. In Deng et al. (2003), the volatiles were collected from green leaves and stems at elevated temperature, i.e., 60°C and the analysis was carried out by using polar stationary phase column. Although Fan and Sokorai (2002) employed a similar SPME fiber to the one we used in the present study, however, the volatiles collection was made at elevated temperature. A number of previous studies showed that collection time, temperature, nature of compound and type of fiber and column are important factors affecting the composition of extracted volatiles from biological samples (Agelopoulos and Pickett, 1998; Deng et al., 2003; Azeem et al., 2013).

Compounds such as (*Z*)-3-hexenol, (*E*)-2-hexenol, and (*Z*)-3-hexenyl acetate are collectively known as green leaf volatiles (GLVs), which form after mechanical damage of plant cells due to enzymatic degradation of cell-wall lipids (Matsui, 2006). These volatile compounds are important constituents of the flavor and aroma of fresh green leaves and impart a characteristic odor referred to as the “green note” (Ul-Hassan et al., 2015). The Desi, Peshawari and Irani cultivars were observed to produce more amounts of GLVs compared to the Hybrid cultivar.

Aldehydes were the most abundant group of volatiles in both HS of fresh leaves and essential oil samples, including 6 and 14 compounds, respectively. (*E*)-2-Decenal was the most abundant compound of all essential oils, except in the Desi cultivar in which the most abundant compound was decanal. In all cultivars the relative abundances of aldehydes were more than 70% of total essential oil, however, the relative composition of different aldehydes were different in four coriander cultivars. In contrast to essential oils the coriander leaves HS aroma of all cultivars consisted of six aldehydes but their relative proportions were far little compared to GLVs. If the GLVs in the HS aroma are ignored, the coriander leaves mainly consist of six aldehydes including (*E*)-2-dodecanal and (*E*)-2-tetradecenal as most abundant compounds. Interestingly these aldehydes were also present in all essential oils in large proportions. In essential oils, the GLVs were observed in trace amounts compared to fresh leaves’ HS aroma, which consisted of about 73–85% of the coriander leaves’ aroma. These compounds are usually formed after mechanical damage of plant cells due to enzymatic degradation of cell-wall lipids (Matsui, 2006). Contrary to the composition of HS volatiles, the essential oils mainly contained aliphatic aldehydes and alcohols, although there are some terpenoids present in trace amounts. Aldehydes in essential oils were mainly come from leaves as indicated by HS analysis but additional aldehydes might have been formed by thermally enhanced oxidation of unsaturated fatty acids (Frankel, 1983; Cao et al., 2020) during distillation process. Subsequently, the aldehydes could be partly reduced to corresponding alcohols. Moreover, the disappearance of 3-hexenyl acetate in essential oils was probably due to degradation during the distillation process. In addition to two major groups of volatiles, i.e., aldehydes and alcohols, five terpenes and one isothiocyanate were exclusively present in the essential oil samples.

In the present study the major compounds in different cultivars essential oils were decanal, (*E*)-2-decenal, (*E*)-2-decenol, 1-decanol, dodecanal, (*E*)-2-dodecenal, and (*E*)-2-tetradecenal, however, their relative abundances were different in different cultivars. The chemical compositions of coriander leaves’ essential oils extracted from Hybrid, Irani and Peshawari cultivars were in agreement to some extent to a study carried out in Kenya (Matasyoh et al., 2009) that described the presence of (*E*)-2-decenal (15.9%) and decanal (14.3%) as most abundant compounds. Whereas the chemical composition of Desi cultivar essential oil was a bit similar to a study conducted in Poland (Nurzyńska-Wierdak, 2013) that showed the presence of decanal (17.2%) as major compound in vegetative stage of coriander leaves essential oil. A previous GC-FID based study from Pakistan reported (*E*)-2-decenal (32.23%), linalool (13.97%), (*E*)-2-dodecenal (7.51%), (*E*)-2-tetradecenal (6.56%) and decanal (1.73%) as major compounds in the coriander leaves’ essential oil (Shahwar et al., 2012), whereas a study from United States conducted in 1990 described (*E*)-2-decenal (46.1%), (*E*)-2-dodecenal (10.3%), (*E*)-2-tetradecenal (5.8%), and decanal (4.4%) as major components of coriander leaves essential oil (Potter and Fagerson, 1990). Though, the major compounds in different studies were same however, their relative proportions were different from each other. The differences in the chemical compositions of coriander from various regions could be due to genetic factors, abiotic stresses, process of oil extraction, variation in climate, altitude, soil composition, and stage of maturity at the time of harvest (Hussain et al., 2008; Priyadarshi and Borse, 2014).

Overall, the chemical composition of essential oils extracted from the Desi and Hybrid cultivars was almost similar except the relative proportion of a few major compounds such as (*E*)-2-decenal, 1-decenol and (*E*)-2-dodecenal. Similarly, the Irani and Hybrid cultivars essential oils composition was found similar with few exceptions whereas Irani and Peshawari were more close to each other. However, the essential oils extracted from the Desi and Peshawari cultivars showed more pronounced quantitative differences. For example, the relative abundance of decanal was higher in the Desi cultivar compared to the Peshawari cultivar, whereas the relative proportion of (*E*)-2-decenal and 2-undecenal were significantly higher in the Peshawari essential oil compared to the Desi essential oil.

DPPH scavenging activity of the coriander leaves’ essential oil was in the range of 6–15%. The essential oil of the Hybrid cultivar exhibited highest DPPH activity compared to the other cultivars. The free radical scavenging activity of Hybrid cultivar leaves essential oil was similar to a previous report that described 20.83% activity (Shahwar et al., 2012), however, other oils exhibited quite low compared to Hybrid and reported data. The MIC of essential oils against pathogenic bacterial strains was in range of 500–1,000 μg/mL. The MIC results were similar to a previous study from Turkey that showed the essential oils from green coriander leaves exhibited MIC 500 μg/mL (Yildiz, 2016). Overall all the tested essential oils showed similar MIC values except Irani and Peshawri cultivars essential oils those showed bit higher MIC against *B. subtilis* and *E. coli*. The minor difference in anti-bacterial activity could be attributed toward synergetic effect of different major and minor components of essential oils.

## Conclusion

The chemical composition of HS aroma of fresh coriander leaves was substantially different from that of essential oils extracted through steam distillation. Qualitative aroma composition of fresh coriander leaves was similar in all cultivars; however, the relative abundance of aroma components was cultivar specific. The HS of the Desi and Peshawari cultivars exhibited a greater presence of green leaf volatiles, giving a characteristic odor referred to as the “green note,” compared to the other cultivars. The Hybrid cultivar was rich in essential oil and showed the highest free radical scavenging activity compared to the other cultivars investigated in this study. Desi and Hybrid cultivar essential oils were found to be most active against all tested pathogenic bacteria.

## Data Availability Statement

The raw data supporting the conclusions of this article will be made available by the authors, without undue reservation.

## Author Contributions

MA, SK, A-KB-K, and GZ: conceptualization. MA, SK, and SS: methodology. MA, A-KB-K, and RA: data analysis. MA and SK: writing—original draft preparation. MA, SK, and RM: writing—review and editing. MA and RM: visualization. MA and GZ: supervision and funding acquisition. MA: project administration. All authors have read and agreed to the published version of the manuscript.

## Conflict of Interest

The authors declare that the research was conducted in the absence of any commercial or financial relationships that could be construed as a potential conflict of interest.

## Publisher’s Note

All claims expressed in this article are solely those of the authors and do not necessarily represent those of their affiliated organizations, or those of the publisher, the editors and the reviewers. Any product that may be evaluated in this article, or claim that may be made by its manufacturer, is not guaranteed or endorsed by the publisher.
